# Icones Obstetricæ; a Series of Sixty Plates Illustrative of the Art and Science of Midwifery in All Its Branches

**Published:** 1843-04-01

**Authors:** 


### ICONES OBSTETRICÆ; A SERIES OF SIXTY PLATES, ILLUSTRATIVE OF THE ART AND SCIENCE OF MIDWIFERY IN ALL ITS BRANCHES. By *A. L. Moreau*, Professor of Midwifery to the Faculty of Medicine, Paris. Edited by *J. S. Streeter*, author of Practical Observations on Abortion, &c. Folio, London, 1842. *Bailliere.*

THIS is certainly one of the most magnificent series of Anatomical Plates which we have ever seen. The drawings are quite admirable, and, indeed, could not be surpassed for accuracy and beauty.

They are from designs by M. *Emile Beau* of Paris, and are exceedingly well lithographed by *Fourquemin.* Many of them are of the full size, and represent different views of the pelvis and its contents, as they appear in nature; others are reduced to one half the natural size.

The first four plates represent the bones of the pelvis, detached or united, in a variety of positions and sections. The fifth one gives a most instructive view of its direction and axes in the erect position, and also of the relative position of the gravid uterus, at the full period, to the brim or inlet; this plate alone is worth a volume of mere description. The following ten plates contain numerous representations of the malformations and deformities to which the pelvis is liable.

By a careful inspection of these, the student will at once perceive how seriously the process of parturition may be interfered with by even a slight deviation from the normal conformation of the osseous structure, and, at the same time, he will learn to appreciate the necessity of making himself thoroughly conversant with the various measurements and diameters of the pelvis, in order to enable him to determine the practicability of delivery, whether natural or instrumental, in such unfortunate cases. True of all things, but doubly true in medical matters, is the Horatian saying—

> Segnius irritant animos demissa per aures,
> Quam quæ sunt oculis subjecta fidelibus, et quæ
> Ipse sibi tradit spectator ————————

A glance at a good drawing often teaches more than listening to a dozen of lectures.

In plates 16, 17, 18, 19, and 20, we have most faithful views, full size, of the pelvis, with its soft linings and contents: the sectional view, in the last one, is especially instructive. Then follow the different sections of the internal genital organs of the adult female, the vagina, uterus, ovaries, &c. In plate 24, there is a graphic representation of the lymphatic vessels of the uterus and its appendages injected, in a woman who died a short time after delivery; and in 25, the nerves of the uterus are well delineated.

Passing over, with a simple notice, several of the following plates, which contain many beautiful drawings of the ovum and fœtus in the successive periods of their development, we come to plate 36, which commences a series of most valuable pictorial illustrations of the mechanism of labour in its various stages.

The relative positions of the head and body of the child with the different parts of the pelvic cavity during the progress of parturition, in natural as well as in irregular presentations, are most faithfully represented: the drawings, we may mention, are half the size of life.

As Mr. *Streeter* very justly observes; "the power should be acquired of seeing with the mind's eye the child through the walls of the uterus. This may be obtained by a careful study of these plates, by habits of reflection, and the aid of a little experience."

The remaining 15 plates illustrate the different operations of practical midwifery, as that of turning* in different presentations of the child, the application of the forceps, &c. &c.

Such is a brief notice of these Icones Obstetricæ, which we hope will become generally known. We are indebted for the introduction of them into this country to a most intelligent practitioner in this metropolis, Mr. *Streeter*, who is already favourably known to the profession by his recent Treatise on Abortion. He has added much to the value of the present work, by appending to most of the plates some useful remarks, in the way of commentary on each subject that is illustrated.

We have much pleasure in selecting a few passages, to enable our readers to judge for themselves of the ability with which the editor has performed his task.

1. *Sanguineous Infiltration of the Labia.*

.. .. .. .. .. .. "The infiltration is commonly confined to one labium, and comes on suddenly during the progress of, or very soon after, the birth of the child. The labium assumes a livid or black colour, and the distention is often excessive. It occasionally ruptures, and the loss of blood has been known to prove fatal.

"More commonly, the internal surface of the labium (unless freely incised, as Dr. *Dewees* practises,) sloughs, and the coagulum, which soon becomes offensive, when exposed to the air, is thrown off, by the suppurative process."

" .. .. .. .. .. .. .. ..

The editor attends a lady, mother of seven children, in whom a swelling appeared at the right inguinal ring, about the seventh month of her first pregnancy. This was pronounced to be a hernia, and a truss was actually applied by a surgeon: it was worn during the pregnancy, and even during labour, but occasioned great distress. The tumor however is a varix, and associated with a dilated and tortuous vein in the right labium, as large as the little finger. The inguinal and labial varices disappear in the unimpregnated state, but regularly return about the middle of each pregnancy."

2. *Protrusion of the Bladder during Labour.*

"A more frequent (than calculus) cause of retarded labour is the protrusion

---

* M. *Moreau*, the editor observes, has illustrated this obstetric operation (version or turning) at great length, devoting no less than eight plates (sixteen figures,) to the elucidation of its performance, a sufficient testimony of its practical worth and importance, as a means of accomplishing delivery. The study of the two principal varieties of version—1, that where the vertex of the child presents; and 2, that where the shoulder presents, will enable the practitioner to conceive and adapt the operation to all those minor varieties which a different position of the child, or an irregular placing of the limbs, may occasion. The successive steps of the operation are all clearly represented, and minutely, though concisely, described."

of the urinary bladder before the head of the fœtus, an accident to which Mr. *Christian* has very ably drawn attention in the 9th vol. of the Ed. Med. and Surg. Journ.

"The bladder may protrude as an elastic tumor under the arch of the pubis, occupying the anterior part of the vagina, or it may be forced down on one of the sides of the pelvic cavity. The remedy is simple—the introduction of the catheter. Dr. *Merriman* has known the tumor punctured with a fatal result, under the idea that it was the head of a hydrocephalic child."

3. *The Bloodvessels of the Uterus; the inefficacy of Compressing the Aorta in Uterine Hæmorrhage.*

"The vascular system of the uterus merits the closest study of the accoucheur, in consequence of the hæmorrhage to which this organ is subject on the separation of the placenta, and from the not unfrequent occurrence of uterine and crural phlebitis. The veins are destitute of valves, and communicate so freely with each other as to present some resemblance to erectile or cavernous tissue. This readily explains the danger of the erect position immediately after delivery, and before the uterine sinuses are securely closed by the muscular and elastic contraction of the uterus, and the formation of coagula within their open orifices.

"As the ovarian arteries supply the part of the uterus where the placenta usually adheres, compression of the aorta, which is only practicable below the origin of these arteries, though recommended by high authority, can exert no other influence in stopping uterine hæmorrhage than by rousing the uterus itself to efficient contraction."

4. *Influence of the Gravid Uterus on the Adjacent Nerves.*

"The cutaneous and muscular branches of the spinal nerves, which are distributed to the lower extremities, merit some attention in an obstetrical point of view, as their functions are often disturbed during pregnancy and parturition. When the gravid uterus sinks down into the pelvis, the irritation and pressure of the cutaneous and anterior crural nerve cause pain and numbness on the fore and outer part of the thigh; when the head is passing through the brim, the obturator nerve suffers compression, and cramp is produced on the inner side of the thigh. As the head advances through the cavity, the sciatic nerve is compressed, and cramp occurs at the back part of the thigh, the calf of the leg and sole of the foot. Occasionally indeed this nerve suffers so much injury as to cause temporary lameness or even partial paralysis."

5. The following interesting remarks on *the Arterial and Venous connection between the Uterus and Placenta* are appended to Plate 30, which gives a view of the internal surface of the uterus, with the placenta adhering to it.

"The tortuous course and abrupt termination of the utero-placental arteries were represented by Dr. *W. Hunter*; and the terminal sinus of the placenta and its free communication with the venous system of the uterus by three or four apertures are figured in this beautiful plate. This utero-placental venous arrangement has been generally altogether overlooked, and the existence of any vascular communication between the uterus and placenta denied by many observers. Its unquestionable existence however proves that there is not only a fœtal, but also a maternal, portion in the human placenta, which is thrown off along with the fœtal portion at birth. * * * * The absolute necessity for this free vascular connexion between the ovum and the parent will be fully recognised when the placenta comes to be generally regarded as the primordial lung, as well as the stomach, of the fœtus. The placenta is in fact not only a nutritive organ, but the analogue of the allantois in the ovum of the bird,—a complex branchial or respiratory organ, operating the same changes on the fœtal blood through the sanguineous medium of the proper uterine circulation, as the gills of aquatic animals produce on their blood through the watery medium in which they live. * * * Comparative anatomy shows that the blood-vessels of the mother are in no instance continuous with those of her offspring; but in all, there is provision made for extensive approximation of the fœtal and maternal vessels. * * * The uterine veins lose their external tunic, and in the placenta retain only their delicate inner coat; the placenta is pierced like a sponge by the transmitted vessels of the uterus (the tortuous arteries of *Hunter*), and the maternal blood circulates freely in a cellular tissue round the tufted vessels of the embryonic portion of the placenta: after which, the blood finds its way into the terminal sinus of the placenta, and is returned to the maternal system by the decidual venous apertures represented in this plate."

### 6. *Management of Foot or Breech Presentations.*

"The frequent occurrence of breech and footling presentations, and the danger to which they expose the life of the child, render their proper management an object of great practical interest. From injudicious attempts to hasten or assist the passage of the nates through the vulva, the child often perishes, the cord being compressed between the head of the child and the parts of the mother. This is owing to the soft parts being badly prepared to admit the speedy passage of the head. To guard against this danger, the nates should always be permitted to pass slowly and unassisted through the external parts so as gradually and perfectly to dilate them, and thereby facilitate the subsequent transit of the head. In this, and indeed in all instances in which a præternatural presentation is suspected, the practitioner should take more than usual care not to rupture the membranes; because, the more relaxed and dilated the soft parts are before they give way, the more favourable will be the result, and a descent of the umbilical cord—a circumstance which occurs far more frequently in feet than in other presentations—will be less likely to take place. When the nates are born, the pulsation of the cord should be minutely watched; and, if the pulsation becomes feeble or ceases, the birth ought to be accelerated by every possible manipulation, lest the child be asphyxiated by the suspension of the respiratory function of the placenta. The delay of even a few minutes is occasionally fatal, where the umbilical circulation is interrupted. When the child is expelled with its abdomen towards the pubes, it should be gently, but sufficiently turned round to direct the vertex into the oblique diameter of the brim, the most favourable position for its passage through the pelvis. The perineum should be carefully guarded."

### 7. *Conduct of an Accoucheur before using any Obstetrical Instruments.*

The following beautiful remarks of the late Dr. *Hugh Ley*, whose loss was so generally regretted a few years ago by his professional brethren, are given from MS. notes of his lectures taken by the Editor in 1822: they bespeak the judicious physician and the kind-hearted man.

"When the symptoms demand the use of instruments, it is right to explain to the friends the necessity and grounds of their employment. This gives them the opportunity of having a consultation, which is always desirable when the circumstances of the case will admit. You should also prepare the mind of your patient by telling her that the time has now arrived for delivering her, that the head sticks in the passage, and that you are going to use *a pair of artificial hands* to fetch it. Shew her first one blade of the forceps, and let her feel its smoothness and satisfy herself that it has no cutting edge, and then the other, but always separately. If the forceps are locked when she first sees them, they suggest to her mind the idea of a cutting instrument like a pair of scissors. You may then apply them gently over her closed hand, taking care not to press the blades too much so as to hurt her. In this manner you may soothe her fears, and, when so explained, they seldom create much alarm. Indeed, you will be much more often urged to apply them when unnecessary, than have their use objected to when required. *Never employ any instrument secretly ;* that is morally wrong, and it is also unwise, because it deprives you of a certain amount of reputation, which always attends the use of instruments, and which may lead to your professional success. I know one individual who dates his first introduction to fashionable practice, from nearly breaking the jaw of a young baronet by the use of the lever, in a case where he now doubts the propriety of his having employed any instrument at all."

In taking leave of this work, we cannot too strongly recommend it to practitioners in general, and more especially to all lecturers on midwifery: the drawings require only to be seen to be appreciated. With the advantage of such a series of plates, and by frequently exercising himself with a female pelvis and a full-grown fœtal head, a student might in six months acquire a really practical knowledge of many of the most difficult emergencies that are apt to occur in the art of midwifery. We are glad to find that Mr. *Streeter* makes repeated reference to the clinical report of Dr. *Collins* of Dublin—a work which, at the time of its publication five or six years ago, we pronounced to be one of consummate interest and value.

